# Neurodevelopment in congenital heart disease: a review of antenatal mechanisms and therapeutic potentials

**DOI:** 10.1038/s41390-025-04360-y

**Published:** 2025-09-11

**Authors:** Amraha Sajid, Raul Chavez-Valdez, April N. Sharp, Divyen K. Shah

**Affiliations:** 1 Barts and the London School of Medicine and Dentistry, Queen Mary University of London, London, UK; 2 Department of Paediatrics, Division of Neonatology, Johns Hopkins University School of Medicine, Baltimore, MD USA; 3 Neonatal Intensive Care Nursery Program, Johns Hopkins Hospital, Baltimore, MD USA; 4 Department of Neurology and Developmental Medicine, Kennedy Krieger Institute, Baltimore, MD USA; 5 Department of Neurology, Division of Pediatric Neurology, Johns Hopkins University School of Medicine, Baltimore, MD USA; 6 Neonatal Intensive Care Unit, Royal London Hospital, Barts Health NHS Trust, London, UK

## Abstract

**Abstract:**

Improvements in treatment and survival of neonates with complex congenital heart disease have led to growing recognition of the associated neurodevelopmental impairments. In this review, we explore the possible antenatal mechanisms of altered brain development in these patients with particular focus on acquired and genetic risk factors. Hypoxia, placental pathology and maternal stress are acquired factors with therapeutic potential. Among the genetic and epigenetic mechanisms, we discuss the influences of angiogenic genes, chromatin modifiers, Wnt and Notch signalling pathways, chromosomal syndromes and apolipoprotein E alleles. Understanding these antenatal risk factors will allow for further development of currently experimental fetal therapies, such as maternal hyperoxygenation.

**Impact:**

- Congenital heart disease has a substantial impact on neurodevelopmental outcome.
- This scoping review identifies and brings together overlapping genetic, environmental and hemodynamic antenatal mechanisms that impact on altered brain development.
- A better understanding of these mechanisms may lead to development of therapeutic potentials to improve neurodevelopment outcomes in this group.

## Introduction

Congenital heart disease (CHD) affects up to 1.35 million newborns every year globally.^1^ As survival rates have increased, long-term adverse neurodevelopmental outcomes have become more apparent and are associated with reduced quality of life in survivors. Impairments in motor performance, language, social functioning, attention and executive function amongst others are well documented deficits in CHD survivors^2^ and are associated with decreased brain volume and white matter abnormalities on MRI.^3,4^ Neurocognitive impairments persist throughout the lifespan and have the most significant long-term impact on quality of life.^5,6^ As many as 65% of children with CHD require remediation services by adolescence and a quarter will receive some form of counseling or psychotherapy.^7^ Adults with CHD have lower average educational attainment, which impacts employment and self-sufficiency, as well as lower rates of marriage and significant partner relationships.^8^

Understanding the risk factors associated with neurodevelopmental impairments in this population has therefore become a priority. The conventional assumption has been that the fetal brain is resilient to hypoxemia and thus that adverse neurodevelopmental outcomes associated with CHD must be related to the postnatal course, such as complications related to surgery, cardiopulmonary bypass and extracorporeal membrane oxygenation (ECMO).^9–11^ This assumption has been challenged over the last two decades with the advent of powerful brain imaging techniques, which have demonstrated cerebral dysmaturity and injury present in the fetus and the newborn before surgical interventions.^12^ Hence, these brain abnormalities may originate from acquired and genetic factors both disrupting brain development and causing injury to the developing brain. Elucidating the antenatal mechanisms by which cerebral abnormalities and associated neurodevelopmental impairments occur may inform novel antenatal therapeutic options with potential to improve long-term outcomes and quality of life.

**Fig. 1:**
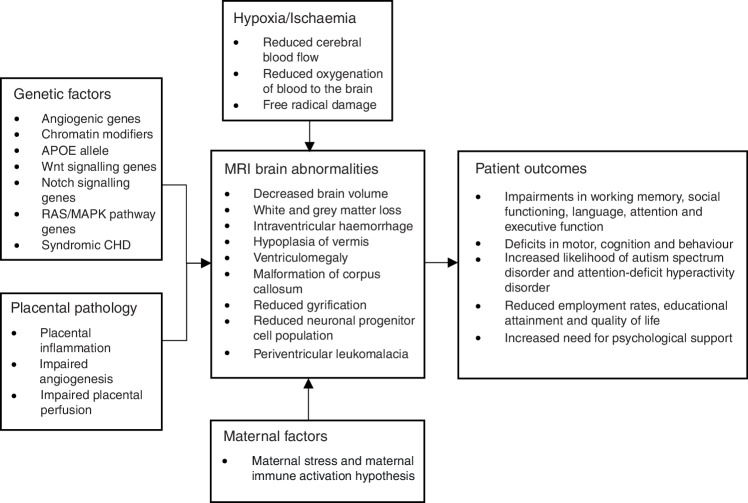
Legend: Summary of the postulated antenatal contributory factors to impaired neurodevelopment, cerebral abnormalities and long-term outcomes of CHD patients. Genetic factors, placental pathology, maternal stress/immune activation, and hypoxia/ischaemia contribute to MRI-detected brain abnormalities (e.g., reduced brain volume, white and grey matter loss, reduced gyrification, and periventricular leukomalacia). These abnormalities underlie patient outcomes including cognitive, motor, and behavioural deficits, increased risk of autism spectrum disorder and ADHD, and reduced quality of life. Arrows (→) indicate directional relationships; bullet points (•) denote specific examples.

This scoping review aims to bring together the current state of understanding to identify knowledge gaps in the field of brain injury in newborns with CHD and to explore potential therapeutic targets. After a brief review of the complex relationship of fetal cardiac physiology and brain development, we will explore in detail the currently described antenatal mechanisms with therapeutic potential. In individual patients, a compounded effect of multiple mechanisms is most likely, given the multifactorial nature of neurodevelopmental impairment in patients with CHD (See Fig. 1).

## Fetal brain development and injury in CHD

Cardiac development comes to near completion around weeks 7–8 of gestation whereas brain development occurs continuously from the third week of embryonic gestation until early adulthood.^13^ The presence of a congenital heart defect can therefore impact the fetal brain for the majority of the gestational period. To briefly review normal fetal physiology, gas exchange occurs in the placenta. Oxygenated blood then travels through the umbilical vein to the brain by flowing from the right atrium to the left atrium via the foramen ovale.^13^ In CHD, structural changes may impact oxygen delivery and perfusion of the fetal brain with some conditions having a greater effect on brain perfusion and oxygenation. For instance, in transposition of the great arteries (TGA), in which the positions of the aorta and pulmonary artery are interchanged, the brain receives less oxygenated blood whilst the more highly oxygenated blood is redirected to the pulmonary system and through the ductus arteriosus to the distal body.^14^ In hypoplastic left heart syndrome (HLHS), the left side of the heart is underdeveloped, leading to oxygenated and deoxygenated blood mixing in the right atrium before being pumped to the lungs and brain. Aortic atresia leads to restricted and retrograde blood flow to the brain, further impairing oxygenation of the cerebral tissue.^15^ In contrast, the structural changes in ventricular septal defect (VSD), the most frequent CHD, may have less consequence on fetal brain perfusion and oxygenation due to less overall hemodynamic impact. More complex cardiac anatomy may lead to greater impacts on cerebral perfusion and oxygenation.

Advances in fetal and neonatal neuroimaging have provided better understanding of the brain alterations associated with CHD. Neuroimaging abnormalities are common in newborns with CHD even prior to surgical interventions.^15^ Microhaemorrhages, germinal matrix and intraventricular hemorrhages, focal and diffuse white matter injury, focal ischemia, and reductions in gray and white matter volume can be seen. Delayed cortical development associated with brain dysmaturity, have been detected in fetal and neonatal imaging.^16^ Using fetal MRI, Rollins and colleagues^17^ have documented a reduction in the size of the intermediate layers and subplate of the subventricular zones (SVZ) prior to 32 weeks gestation in human fetuses prenatally diagnosed with HLHS and TGA. The SVZ contains pre-myelinating oligodendrocytes (Pre-OL), which are exquisitely vulnerable to hypoxia.^18,19^ A decrease in neural progenitor cells, impaired development of the SVZ and reduced gyrification and cortical gray matter volume have been elegantly demonstrated in an experimental CHD model.^20^ These brain abnormalities are associated with long-term neurodevelopmental impairments.^21,22^ Identifying and understanding the mechanisms of brain dysmaturity and injury could therefore aid development of therapeutic targets to improve neurodevelopmental outcomes.

## Potential mechanisms of altered brain development in CHD

### Acquired factors

The environment in which the fetus develops results from a complex interplay of maternal and fetal physiology. Alterations in these factors may impact fetal brain development and maturation.

### Impaired cerebral oxygenation and perfusion in complex CHD

We have already explored how abnormal cardiac physiology may reduce fetal brain perfusion and oxygenation. The mechanisms through which hypoxia alters brain development and causes injury in CHD are not fully characterized, but may involve effects on Pre-OL, which are susceptible to hypoxic-ischemic injury.^23^ Free radicals are produced in response to the hypoxic environment and mediate oligodendrocyte cell death.^24^ The reduction in this cell population then results in disrupted myelination, decreased brain volume and reduced neuronal density in models of intrauterine chronic hypoxia^25^ as well as intrauterine growth restriction^26–28^ and smaller brain size associated with reduced cerebral oxygen consumption in CHD human foetuses.^29^ Interventions to decrease chronic hypoxia and its downstream effects are potential therapeutic targets to mitigate CHD-associated brain injury.

### Placental pathology

The association of placental pathology with neurodevelopment in CHD patients is well recognised.^30^ Both placental structural development and placental function can be disrupted in CHD.^31,32^ Increased severity of placental abnormality has been shown to correlate with decreased brain volume in severe CHD when placental pathology scores have been calculated using parameters including placental weight, umbilical cord insertion, umbilical cord coiling index, placental maturation, maternal vascular malperfusion and inflammation or reactive changes.^33^ Fifty-seven percent of CHD patients, especially those with aortic obstructions, were found to have placental abnormalities, such as infarction, thrombosis, and villi immaturity, which were associated with haemodynamic changes and hypoxia in the fetus.^34,35^ In another study, 78% of placentas in the CHD group were found to have placental pathology compared to 28% in the control group. Maternal and fetal vascular malperfusion lesions, such as placental hypoplasia, infarct, thrombosis, and chorangiosis were only present in placentas complicated by CHD.^36^ They also identified that head circumference at birth was significantly lower in CHD cases compared to controls.^36^ Using advanced MRI imaging of the placenta, global perfusion has been demonstrated to be lower in CHD pregnancies than in controls.^37^ While this association is compelling, current data do not establish a causal relationship between placental abnormalities and CHD.

Placental inflammation is documented in 35% of pregnancies with fetal CHD^33^ which is higher than that reported in the general population.^38^ Chronic placental inflammation and impaired maturation are the most common placental abnormalities found in fetuses with aortic obstructions.^34^ The association of maternal inflammation during pregnancy and ND outcomes in newborns is well recognised;^39^ however, the role that placental inflammation may play in abnormal heart development in the fetus remains unclear. In a murine study, placental inflammation impaired cardiac tissue structure development, the effects of which continued into the postnatal period.^40^ This was proposed to be mediated by placental inflammatory monocytes migrating to the embryonic heart. Supporting placental function and preventing or treating placental inflammation could potentially reduce acquired brain injury in CHD.

### Maternal stress

Other maternal factors may have an indirect impact on the brain development of fetuses diagnosed with CHD. Up to 50% of parents of CHD infants encounter stress requiring clinical support for up to a year after diagnosis.^41,42^ Maternal stress in particular, which is defined by diagnoses of anxiety and depression measured by standardised tools as well as the need for clinical support, may contribute to neurodevelopmental outcomes. The ‘maternal immune activation hypothesis’ suggests that fetal exposure to maternal immune responses impacts neurodevelopment through a variety of pathways, including exposure of the fetal brain to cytokines.^43^ Alterations in the maternal hypothalamic-pituitary-adrenal axis, such as abnormal transfer and deactivation of cortisol from mother to fetus, may also impact neurodevelopment.^44–46^ Maternal stress, regardless of the presence of CHD, is linked to thinner cerebral cortex^47^ and alterations to the amygdala and the microstructure of the brain.^48,49^ However, in those foetuses with CHD, maternal stress and anxiety were also associated with smaller hippocampal and cerebellum volumes.^42^ It is important to note that prenatal diagnosis of CHD likely contributes to increased maternal/parental stress, but prenatal diagnosis is also associated with improved neurodevelopmental outcomes, highlighting the complex interplay of antenatal mechanisms of brain injury associated with CHD.^50^ Continuing to optimize parental supports from the time of CHD diagnosis and continuing throughout the medical course will be important for both patients and their families and may have therapeutic implications for long-term neurodevelopment.

## Genetic factors

A genetic disorder or clinical syndrome is found in 20 to 30% of CHD, and neurodevelopmental outcomes are most consistent with the underlying genetic disorder.^51^ Fetuses with CHD associated with a genetic or syndromic diagnosis, such as DiGeorge syndrome, CHARGE syndrome, VACTERL association, and Noonan syndrome, have been shown to have a smaller head circumference compared to fetuses with isolated CHD, those without genetic variants, extracardiac malformations or intrauterine growth restriction.^52^ Three types of genetic influences associated with altered brain development in CHD are discussed below: (1) single-gene variants and related syndromes, (2) chromosomal syndromes and (3) genetic polymorphisms. Variants in candidate genes implicated in both cardiac and brain development include angiogenic genes, those encoding for chromatin modifiers and Wnt, Notch and Ras/Mitogen Activated Protein Kinase (RAS/MAPK) signaling pathways. Chromosomal syndromes that include CHD, such as Down syndrome and DiGeorge syndrome, have the highest incidence of neurodevelopmental impairments. Lastly, polymorphisms of genes, such as APOE, act as genetic modifiers of neurodevelopmental risk. Improved understanding of how these genetic changes lead to both cardiac malformations and neurodevelopmental impairments may provide disease-specific therapeutic targets.

### Angiogenic genes

Altered expression of angiogenic genes such as the placental growth factor (PIGF), soluble FMS-like tyrosine kinase-1 (sFlt-1), and vascular endothelial growth factor-A (VEGF-A) have been implicated in disrupted cardiovascular and cerebrovascular development.^53^ While PIGF is reduced in pregnancies where the fetus had a major, isolated cardiac defect, both VEGF and sFlt-1 are increased in cord blood of fetuses prenatally diagnosed with CHD, compared to controls.^54^ The dysregulation of the VEGF pathway has been associated with malformation of the endocardial cushion and cardiac septation, as well as anomalies in the outflow tract and aortic arch.^52^ These genes have also been linked to smaller head circumference at birth and reduced fetal growth.^53^ Higher expression of anti-angiogenic marker, sFlt-1, has been noted in the frontal cortex and in the basal ganglia of fetuses with CHD and associated with decreased blood vessel growth.^54^ CHD pregnancies have reported decreased levels of serum maternal growth factor^54^ and dysregulation of leptin in HLHS patients,^55^ both of which are angiogenic factors.

### Chromatin modifiers

#### Chromatin-modifying genes

Chromatin-modifying genes are responsible for the regulation of gene expression and are highly expressed in genes involved in both heart and brain development. In a large cohort of 1200 CHD patients, 25 de novo loss of function variants in chromatin modifying genes with a 5.3-fold higher prevalence than expected were identified.^56^ Of 69 gene variants in both neurodevelopmental disorders and CHD, 19 were chromatin modifiers.^56^ These 69 genes were also in the top quartile of expression for both heart and brain development, suggesting pleiotropism. Another study of 2871 CHD probands found that 87% of patients with de novo variants in chromatin-modifying genes had neurodevelopmental disabilities (NDD) at enrollment and accounted for 2.3% of proband CHD cases.^57^ At least 38 chromatin-modifying genes were estimated to contribute to CHD.^57^ Syndromes such as Kabuki, CHARGE and CDK13-related syndrome are due to alterations in chromatin modifiers KMT2D, CHD7 and CDK13 respectively.^58,59^ CHD7 and KMT2D, especially, have been implicated. CHD7 plays a role in the development of the cardiac outflow tract and of inhibitory neurons.^60^ (See Table 1)

**Table 1:** Summary of the cardiovascular defects and neurologic abnormalities noted in syndromes.

*Syndrome*		Genetic Modifiers	Congenital heart defect	Brain Abnormalities
*Chromosomal* *Abnormalities*	Down Syndrome	RUNX1, DYRK1A	• Atrioventricular septal defect • Ventricular septal defect^74^	• ↓ NPC proliferation, myelination, and spinal cord motor neurons • ↑ astrocytic density, microglia activity • Changes in synaptic density^116^
	DiGeorge Syndrome	TBX1, DGCR8, HIRA	• Tetralogy of Fallot • Truncus Arteriosus • Narrowing of Aortic Arch • Coarctation of the Aorta^86^	• White matter hyperintensities • Septum Pellucidum defects • White and gray matter abnormalities • Decreased cerebellum volume^117^
*RAS-opathies*	Costello Syndrome	HRAS	• Hypertrophic Cardiomyopathy^76^	• Ventricular dilatation • Brain atrophy • Chiari malformation • Syringomyelia^118^
	Cardiofaciocutaneous syndrome	BRAF, HRAS, MAP2K1, MAP2K2	• Pulmonary stenosis • Hypertrophic Cardiomyopathy • Atrial Septal Defects^76^	• Ventriculomegaly • Perivascular spaces • Abnormal myelination • Hydrocephalus^119^
*Chromatin regulation disorders*	Kabuki Syndrome	KM2TD	• Coarctation of the aorta • Atrial and Ventricular Septal Defects • HLHS • Outflow defects^120^	• Decreased gray matter volume • Reduced hippocampal and dentate gyrus volume • Reduced cerebral blood flow^121^
	CHARGE syndrome	CHD7	• Conotruncal Defects • Atrioventricular septal defects • Outflow defects • Septal defects • Patent Ductus Arteriosus^122^	• Brainstem and cerebellar hypoplasia • Ventriculomegaly^123^
	CDK13-related syndrome	CDK13	• Atrial Septal Defects • Ventricular Septal Defects • Pulmonary Valve Abnormalities • Hypoplastic Pulmonary Artery • Bicuspid aortic with aortic stenosis^124^	• Dysgenesis of corpus callosum • Periventricular gliosis • Decreased white matter volume • Spinal Cord Syrinx • White matter abnormalities • Cerebellar Tonsillar Abnormalities^125^

#### Chromatin-modifying enzymes

Disruption of chromatin-modifying enzymes such as histone methyltransferases and demethylases are also linked to CHD and NDD.^61^ Trithorax and polycomb group of proteins are involved in regulating the enzymes H3K4 and H3K27 methyltransferases, respectively which regulate gene expression via chromatin remodeling. Several genes associated with these two protein groups were present in both CHD and NDD patients. Furthermore, knock out studies have shown that absence of these genes results in defective development of the brain and heart.^61^

### Signaling pathways

Of the 69 gene variants in both CHD and NDD noted by Homsy et al.^56^ 32 were transcriptional regulators, including genes involved in Wnt and Notch signaling. These pathways are responsible for determining the fate of cells, regulating developmental processes, and the homeostasis and regeneration of tissue via gene transcription and cell-cell communication.^62,63^ The implicated Wnt genes include CTNNB1, DVL3 and LRP5 whilst EP300 and NOTCH1 are genes involved in Notch signaling.^56^

#### Wnt signaling

The Wnt pathway is heavily involved in transcription regulation and is involved in the development of the embryonic heart via cell adhesion, cardiomyocyte differentiation, valve formation, septation, and development of both the cardiac conduction system and outflow tract.^64^ It is also involved in brain maturation via formation of the neural tube, proliferation of neural precursor cells, dendritic development, and synaptogenesis. Copy number variations in Wnt-related genes have been noted to be significantly enriched in CHD.^65^ Wnt gene mutations are also associated with neuropsychiatric disorders by altering axon guidance.^64^

#### Notch signaling

The Notch signaling pathway is involved in intercellular communication between multiple organ systems, cell development, and differentiation.^66^ It is specifically involved in triggering the heart tube to loop in embryonic development as well as the specialisation of cells, signaling them to differentiate into cardiac cells, and is therefore vital for cardiac development.^67^ Peak Notch signaling activity has also been found to occur at the onset of neurogenesis during early cortical development, and the pathway is also involved in the development of astrocytes in later phases.^68^ NOTCH1 was identified as one of the 21 gene variants found de novo in patients with CHD. Pathogenic NOTCH1 variants have been found to cause heart lesions in both isolated and syndromic CHD.^69^ They are also found to be significantly enriched in families with both left ventricular outflow CHD and Tetralogy of Fallot. JAG1, which is also involved in the Notch pathway, has been implicated in both syndromic and non-syndromic CHDs.^70^

#### Ras/mitogen activated protein kinase (RAS/MAPK) pathway

The RAS/MAPK pathway is involved in the regulation of cell proliferation, differentiation, migration and survival.^71^ This pathway is vital for both cardiac and brain development and has been implicated in syndromes which affect both systems, such as Costello and cardiofaciocutaneous (CFC) syndromes.^72,73^ The RAS pathway is involved in neurogenesis, myelination, differentiation of oligodendrocytes and development of glial cells.^74^ RASopathies (syndromes caused by pathogenic variants in the RAS/MAPK pathway), often present with developmental delay, neurocognitive impairment, and structural abnormalities, due to disruption of early neurodevelopmental processes.^75^ Those with variants in the HRAS gene may develop Costello Syndrome, which is associated with pulmonary valve stenosis, neurodevelopmental delay, structural abnormalities in the brain and decreased intellectual ability. Similarly, mutations in BRAF, HRAS, MAP2K1 and MAP2K2 can cause CFC syndrome, which manifests with a variety of CHDs such as pulmonary stenosis and atrial septal defects.^76^ Patients with CFC syndrome also have neurological deficits in motor, cognition, behavior, and coordination as well as developmental delay.^76^

### CHD in chromosomal syndromes

Aneuploidies of chromosome 21, 18, and 13, as well as deletions in chromosome 22, manifest as syndromes with both congenital heart defects and neurodevelopmental impairment. In this review, we focus on the most common syndromic CHD conditions: Trisomy 21 (Down syndrome) and 22q11.2 Deletion Syndrome (22q11DS) (commonly known as DiGeorge syndrome).

#### Trisomy 21

Over 40% of individuals with complete or partial Trisomy 21 are born with heart defects such as atrioventricular septal defect or ventricular septal defect.^77^ Trisomy 21 patients have a spectrum of neurodevelopmental impairments involving motor, language, cognitive, and adaptive/social domains. Genetic factors are thought to predispose these individuals to adverse neurodevelopmental outcomes via dysregulation of the RUNX1 transcription factor and the dual-specificity tyrosine-phosphorylation-regulated kinase 1 A (DYRK1A) gene.^74^ RUNX1 is involved in regulating neurological development by promoting proliferation and differentiation of several neuronal populations and was found to be hypermethylated in patients with Trisomy 21 and CHD.^78,79^ DYRK1A is involved in neuronal migration and the development of dendrites, neural progenitors, and synapses.^80^ Overexpression of DYRK1A, as seen in Trisomy 21, inhibits proliferation of neural progenitors and causes reduced neurogenesis.^81^ Smaller brain and cerebellar volumes measured by cerebral MRI have been reported in individuals with Trisomy 21.^82^

#### 22q11.2 deletion syndrome

Microdeletions in chromosome 22 are associated with DiGeorge syndrome. These patients can present with CHD, neurological impairment, immunodeficiency, facial dysmorphisms and hypoparathyroidism. These abnormalities are thought to be related to alterations in the transcription factor TBX1 causing altered development of the pharyngeal arches and pouches in embryos.^83^ TBX1 is heavily involved in the development of the cardiac outflow tract, and isolated TBX1 mutations have been implicated in neuropsychiatric conditions that are also present in DiGeorge syndrome.^84,85^ Seventy-five percent of patients with DiGeorge syndrome develop CHD, including Tetralogy of Fallot, truncus arteriosus and aortic arch abnormalities.^86^ DiGeorge syndrome also presents with neurodevelopmental disorders from early infancy, including autism spectrum disorder, attention-deficit hyperactivity disorder, and cognitive impairment.^87^

In these patients, the number of bases involved in the chromosome 22 microdeletion is inversely related to cortical brain surface area.^88^ Cerebral structure abnormalities have also been noted in adults with DiGeorge, including cerebral and cerebellar atrophy, ventricular enlargement, midline anomalies and the presence of bright white matter foci on T2 MRI imaging.^89^

Additional variants in candidate genes involved in both brain and heart development in patients with DiGeorge syndrome include HIRA and DGCR8. HIRA is a protein involved in histone production and proliferation of neural progenitor cells. In rodents, the lack of HIRA has been noted to affect neurogenesis and dendritogenesis, resulting in smaller corpus callosum and hippocampal regions.^90^ Haploinsufficiency of HIRA also results in developmental heart defects in cultured tissue.^91^ Defective DGCR8, an RNA-binding protein contributes to neuropsychiatric conditions in patients with DiGeorge,^92^ while also leading to impaired cardiomyocyte differentiation and consequently dilated cardiomyopathy.^93^

### Genetic polymorphisms

#### APOE

Lipoproteins pack lipids into water soluble form to allow their transport in the blood stream. Apolipoprotein E (APOE) is also involved in regulation of cholesterol metabolism and neuronal resiliency in the brain.^94^ Polymorphisms of APOE have been implicated in impaired neurodevelopmental outcomes in congenital heart disease. The APOE allele ε2, which has been associated with dyslipidaemia,^95^ has been of interest in relation to neurological outcomes in CHD.^96^ APOE ε2 has been shown to be associated with reduced head circumference in CHD patients who had undergone surgery as infants.^97^ A cohort of patients who had undergone cardiopulmonary bypass within 6 months of birth were found to have lower neurological outcome scores, compared to those with ε3 and ε4 alleles, at age one, independent of their type of cardiac defect, ethnicity or socioeconomic status.^96^ After adjusting for pre- and post-operative variables, follow-up studies found that carriers of APOE ε2 were more likely to have impaired social interactions and behavioral problems,^98^ as well as lower psychomotor development index scores at 14 months age.^99^

## Therapeutic potentials

Understanding early risk factors for abnormal brain development and brain injury in CHD is essential for developing timely interventions with the potential to reduce neurodevelopmental impairments and improve long-term quality of life. Early diagnosis is the first important step. Antenatal diagnosis of TGA and single-ventricle disorders has been associated with lower rates of perioperative brain injury compared to those diagnosed postnatally,^50^ potentially related to better delivery planning and cardiac stabilization. Fetal interventions are the next potential therapeutic target. The role of fetal cardiac interventions, such as aortic valvuloplasty and atrial septostomy,^100^ in improving cardiac and neurologic outcomes in CHD patients is not known at present. Early and comprehensive genetic testing after diagnosis of CHD will help identify conditions that may be amenable to targeted treatments, such as RASopathies (alteration in RAS/MAPK pathway). Treatment with MEK inhibitors in children with Noonan syndrome has shown improvement in cardiac pathology.^101^ In addition, preclinical studies have shown that MEK inhibition can reverse learning and memory deficit in Noonan Syndrome mouse models.^102^ However, neurological effects in human populations require further exploration. Given the many genetic influences on CHD already discussed, additional disease-specific treatments may be possible as their mechanisms continue to be studied.

Acquired risk factors related to the fetal environment may provide more immediate therapeutic targets. Since antenatal diagnosis of CHD is associated with increased maternal stress, which may adversely affect brain development, increased early screening for maternal psychological distress, integrated prenatal mental health support, and targeted early psychological interventions may have long-term benefits for both mothers and children with CHD. Support programmes and education to reduce risk factors for maternal inflammation, such as smoking, obesity, and diabetes, are important for general maternal and fetal health and may have additional benefit for fetuses with CHD.

Specific therapies to target white matter injury and brain dysmaturity are currently under investigation. Maternal hyperoxygenation is being studied clinically as a means to mitigate the effects of fetal hypoxemia related to CHD and will be explored in more detail.^103^ There is also preliminary experimental evidence that tetrahydrobiopterin supplementation may ameliorate injury to Pre-OL and consequently reduce hypomyelination in CHD patients.^104^ Depletion of tetrahydrobiopterin in hypoxic fetal brains is hypothesized to increase production of peroxynitrite leading to increased reactive oxygen species, which are damaging to Pre-OL,^105^ but this intervention remains preclinical at this time.

### Maternal hyperoxygenation

Studies using both acute and chronic maternal hyperoxygenation (MH) have suggested improvement on heart and brain development of CHD foetuses. Depending on the study, acute MH refers to a short (6-20 minutes) single administration of oxygen, whereas chronic MH refers to multiple administrations of oxygen given for a number of hours for several days.^103,106^ Cerebrovascular resistance (CR), as reflected by fetal middle cerebral artery pulsatility index, is increased in response to acute MH in HLHS fetuses over 28 weeks gestation.^107^ Fetuses with lower initial CR demonstrated the greatest increase after treatment, perhaps reflecting a greater return to normal cerebral blood flow. Cerebral vasodilation is postulated to occur as part of the compensatory ‘brain-sparing’ mechanism in response to chronic hypoxia; therefore, an increase in CR may indicate that MH reduces the need for this autoregulatory mechanism by increasing oxygen delivery to the brain. Foetuses with single-ventricle physiology, but not two-ventricle physiology, exposed to MH showed an increased blood-oxygen-level-dependent (BOLD) MRI signal in the placenta and brain compared to healthy foetuses.^108^

In addition to impacting cerebral perfusion, both acute and chronic MH studies have found that hyperoxygenation increases pulmonary blood flow,^109–111^ improves left ventricular filling^112^ and increases mitral valve, ascending aorta and aortic isthmus diameters.^109,113,114^ Chronic administration of 45% FiO_2_ for 2 × 3 h a day was associated with increased birth weight and higher strain rate of both ventricles indicating improved ventricular function and ventricular output.^113,114^ While promising, MH remains experimental at this time. One chronic MH study reported significantly reduced biparietal head growth and smaller head circumference z-scores at 6 months in those exposed to chronic MH in utero.^115^ The mechanism for this is unknown and further studies to assess its safety and potential risks are required. Clinical trials are currently underway to explore the viability of this potential therapy.

## Summary

In conclusion, the neurodevelopmental impairments associated with altered brain development in CHD patients have a significant impact on quality of life. Cerebral abnormalities can be detected in these patients during fetal development. Understanding the antenatal mechanisms of brain injury and neurologic dysfunction is key to developing interventions that will improve long-term function and quality of life for CHD survivors. Acquired and genetic mechanisms are well-described, but not yet fully understood. Ongoing, detailed exploration of how these mechanisms influence altered cardiac and brain development will be vital for discovery and optimization of therapeutic potentials.
